# Engineering of CRISPR-Cas12b for human genome editing

**DOI:** 10.1038/s41467-018-08224-4

**Published:** 2019-01-22

**Authors:** Jonathan Strecker, Sara Jones, Balwina Koopal, Jonathan Schmid-Burgk, Bernd Zetsche, Linyi Gao, Kira S. Makarova, Eugene V. Koonin, Feng Zhang

**Affiliations:** 1 Howard Hughes Medical Institute, Cambridge, USA; 2 Broad Institute of MIT and Harvard, Cambridge, MA 02142 USA; 3 McGovern Institute for Brain Research, Department of Biological Engineering, Massachusetts Institute of Technology, Cambridge, MA 02139 USA; 4 Department of Brain and Cognitive Sciences, Department of Biological Engineering, Massachusetts Institute of Technology, Cambridge, MA 02139 USA; 5 Department of Biological Engineering, Massachusetts Institute of Technology, Cambridge, MA 02139 USA; 6 National Center for Biotechnology Information, National Library of Medicine, National Institutes of Health, Bethesda, MD 20894 USA

## Abstract

The type-V CRISPR effector Cas12b (formerly known as C2c1) has been challenging to develop for genome editing in human cells, at least in part due to the high temperature requirement of the characterized family members. Here we explore the diversity of the Cas12b family and identify a promising candidate for human gene editing from *Bacillus hisashii*, BhCas12b. However, at 37 °C, wild-type BhCas12b preferentially nicks the non-target DNA strand instead of forming a double strand break, leading to lower editing efficiency. Using a combination of approaches, we identify gain-of-function mutations for BhCas12b that overcome this limitation. Mutant BhCas12b facilitates robust genome editing in human cell lines and *ex vivo* in primary human T cells, and exhibits greater specificity compared to *S. pyogenes* Cas9. This work establishes a third RNA-guided nuclease platform, in addition to Cas9 and Cpf1/Cas12a, for genome editing in human cells.

## Abstract (web summary)

The Cas12b family of CRISPR nucleases has been underutilized in mammalian cells due to the high temperature requirement of known members. Here the authors engineer BhCas12b to overcome this limitation for robust and specific genome editing applications in human cells.

## Introduction

**Fig. 1:**
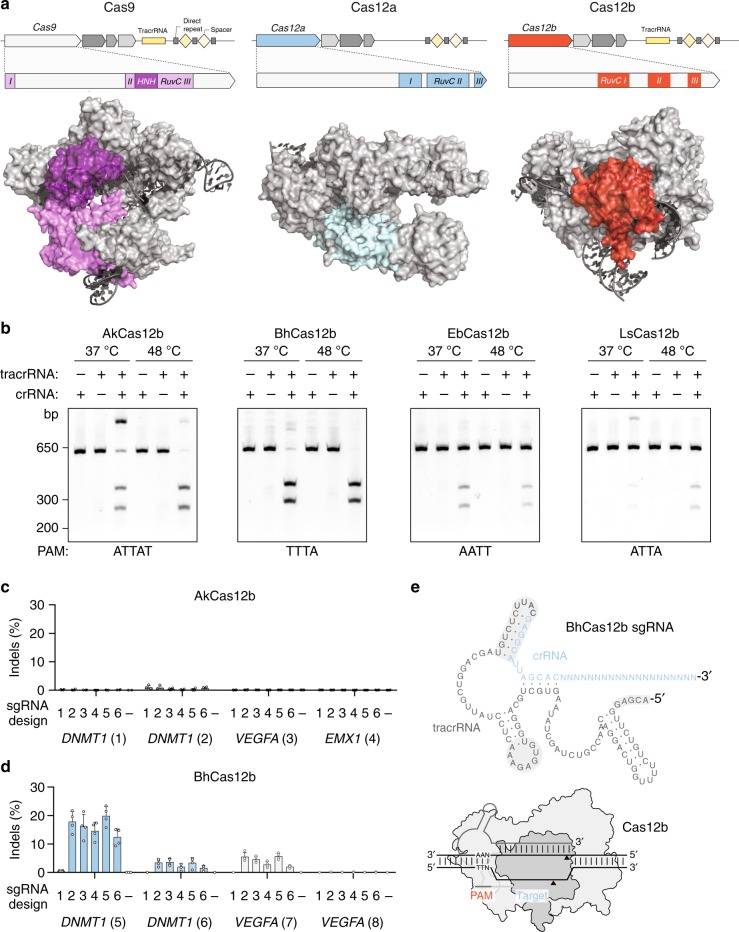
Identification of mesophilic Cas12b nucleases. **a** Locus schematics and protein domain structure highlighting the differences between Cas9, Cas12a, and Cas12b nucleases. Crystal structures of SpCas9 (PDB:4oo8 [10.2210/pdb4OO8/pdb]), AsCas12a (PDB:5b43 [10.2210/pdb4OO8/pdb]), and AacCas12b (PDB:5u30 [10.2210/pdb5U30/pdb]). **b** In vitro reconstitution of Cas12b systems with purified Cas12b protein and synthesized crRNA and tracrRNA identified through RNA-Seq. Reactions were carried out at the indicated temperatures for 90 min and 250 nM Cas12b protein. **c, d** AkCas12b and BhCas12b indel activity in 293T cells with six sgRNA variants. Error bars represent s.d. from *n* = 4 replicates. See Supplementary Fig. 3b, c for sgRNA sequences. **e** Schematic of BhCas12b sgRNA design 1 with gray shading highlighting the location of changes to the guide design (see Supplementary Fig. 3 for exact sequences of guide design variants 2–6). Source data are provided as a Source Data file

Enzymes from the prokaryotic clustered, regularly interspaced short palindromic repeats and CRISPR-associated protein (CRISPR–Cas) systems have been harnessed as reprogrammable and highly specific genome editing tools^1,2^. Current genome editing technologies have focused on class 2 CRISPR–Cas systems, which contain single-protein effector nucleases for DNA cleavage. However, only two families of class 2 nucleases have been harnessed for genome editing in human cells to date: Cas9^3,4^, a dual-RNA-guided nuclease which requires both CRISPR RNA (crRNA) and tracrRNA^5^ and contains both HNH and RuvC nuclease domains^6,7^, and Cas12a^8^, a single-RNA-guided nuclease which only requires crRNA and contains a single RuvC domain. Here, we focus on a third family of class 2 effector, Cas12b, a dual-RNA-guided nuclease containing a single RuvC domain and requiring both crRNA and tracrRNAs^9,10^ (Fig. 1a). Although Cas12b proteins are often smaller than Cas9 and Cas12a and therefore attractive from the standpoint of intracellular delivery via viral vectors, the best characterized Cas12b nuclease from *Alicyclobacillus acidoterrestris* (AacCas12b) exhibits optimal DNA cleavage activity at 48 °C, precluding its use in mammalian cells^9^. We sought to identify Cas12b family members that would be active at lower temperatures and thus could be adapted for human genome editing.

## Results

### Identification of mesophilic Cas12b nucleases

A BLAST search of the updated sequence databases using previously detected Cas12b proteins as queries identified 27 members of the Cas12b family that are encoded within type V–B loci. The type V–B systems are widely scattered among bacteria, and topology of the phylogenetic tree of Cas12b generally does not follow the bacterial taxonomy, suggestive of extensive horizontal mobility. We chose 14 uncharacterized Cas12b genes spanning the diversity of the computationally identified candidates for experimental study (Supplementary Fig. 1a), avoiding previously described members and those from recognized thermophiles. All known class 2 DNA-targeting CRISPR–Cas nucleases require a protospacer-adjacent motif (PAM)^6,8^ for DNA cleavage, and the initial characterization of the Cas12b family revealed a PAM on the 5′ side of the target site^9^. To confirm that each of the identified loci are functional CRISPR–Cas systems and to identify their PAMs, we expressed a human codon-optimized Cas12b with their natural flanking sequence in *E. coli* and challenged transformed cells with a randomized 5′ PAM library followed by deep sequencing (Supplementary Fig. 1b,c). We detected depletion in 4 of the 14 tested Cas12b systems (AkCas12b, BhCas12b, EbCas12b, and LsCas12b), indicating functional DNA interference in a heterologous host. Depleted PAMs were T-rich at positions 1–4 bp upstream of the protospacer, consistent with the preferences observed for previously studied Cas12b members^10^. We performed small RNA-Seq on *E. coli* lysates to identify the required RNA components and found putative tracrRNA mapping to the region between Cas12b and the CRISPR array (Supplementary Fig. 2a–d).

To biochemically characterize Cas12b, we tested for in vitro DNA cleavage activity of purified Cas12b proteins with corresponding tracrRNA and crRNA components (Fig. 1b, Supplementary Fig. 2e). We observed only minimal activity with EbCas12b and LsCas12b; however, both AkCas12b and BhCas12b exhibited strong cleavage at 37 °C, warranting further investigation in human cells. Given that the tracrRNA and crRNA for Cas9 can be fused to form a single-guide RNA (sgRNA)^11^ to simplify delivery, we explored whether sgRNAs can be designed for both AkCas12b and BhCas12b and found that they supported DNA cleavage activity in vitro (Supplementary Fig. 2f). We then transfected 293T cells with plasmids expressing NLS-tagged Cas12b and sgRNA driven by a U6 promoter and measured nuclease activity through the formation of insertion or deletion (indel) mutations by targeted deep sequencing. Indels were detected for both Cas12b proteins, but the rates were below 1% (Fig. 1c, d). To increase efficiency, we tested the effect of changes in the sgRNA scaffolds by altering the tracrRNA and crRNA linkage, removing hairpin mismatches, and modifying the 5′ start site and spacer length (Fig. 1c–e, Supplementary Fig. 3). Although alterations in the AkCas12b sgRNA had little effect, a 5-nt truncation on the 5′ end of the BhCas12b sgRNA substantially improved activity (up to 30-fold) across multiple targets (sgRNA design 2).

### Rational engineering of BhCas12b

**Fig. 2:**
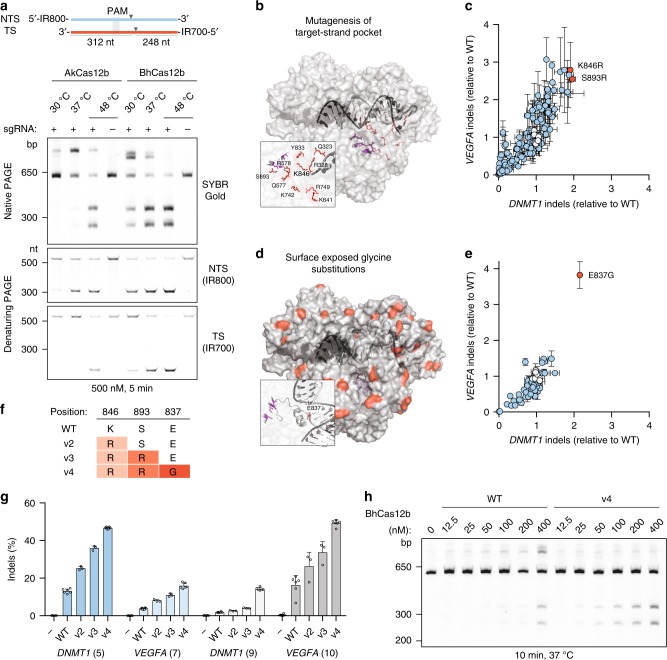
Rational engineering of BhCas12b. **a** In vitro Cas12b reactions with differentially labeled DNA strands. A slower migrating product is observed during native PAGE separation and separation by denaturing PAGE reveals a preference for AkCas12b and BhCas12b to preferentially cut the non-target strand at lower temperatures. **b** Location of 10 of the 12 tested residues in the pocket between the target strand and the RuvC active site (purple). BhCas12b residues are highlighted in the structure of the highly similar BthCas12b (PDB: 5wti [10.2210/pdb5WTI/pdb]). **c** Indel activity of 176 BhCas12b mutations at *DNMT1* (target 5) and *VEGFA* (target 7) normalized to wild type (gray circles). Error bars represent s.d. from *n* = 2 replicates. **d** Location of surface-exposed residues mutated to glycine. **e** Indel activity of 66 BhCas12b mutations at *DNMT1* (target 5) and *VEGFA* (target 7) normalized to wild type (gray circles). Error bars represent s.d. from *n* = 2 replicates. **f** Summary of BhCas12b hyperactive variants. **g** Indel activity of BhCas12b variants at four target sites. Error bars represent s.d. from *n* = 3–6 replicates. **h** In vitro cleavage with increasing concentrations of BhCas12b WT and v4 variant. Gel is a representative image from *n* = 2 experiments. Source data are provided as a Source Data file

We frequently observed a slower migrating band during gel electrophoresis of in vitro cleavage reactions, most notably, with AkCas12b, which suggested that Cas12b can nick double-stranded DNA (dsDNA) substrates (Fig. 1b). Reactions with differentially labeled DNA strands revealed that AkCas12b and BhCas12b preferentially cut the non-target strand, and that this behavior is more pronounced at lower temperatures (Fig. 2a). As the inability to cleave the target strand reduces the potential of BhCas12b as an effective nuclease for genome editing, we sought to address this limitation through protein engineering.

In contrast to the flexible non-target strand, we reasoned that the target strand might be poorly accessible to the RuvC active site of BhCas12b. A crystal structure of a supplementary target strand in AacCas12b revealed several key residues that contact DNA in the pocket between the guide RNA:DNA duplex and the RuvC active site^12^, and we hypothesized that altering the properties of this pocket in BhCas12b might improve target-strand accessibility and DNA cleavage. We mutated 12 BhCas12b residues identified through alignments with AacCas12b, residues which were also conserved in the structure of the nearly identical Cas12b from *Bacillus thermoamylovorans* (BthCas12b)(BthCas12b also exhibits activity in cells, but with lower efficiency than BhCas12b [Supplementary Fig. 4a])^13^. We measured indel activity at two target sites with a total of 176 BhCas12b single mutants and found increased activity with several mutations, including K846R and S893R, which exhibited additive effects as a double mutant (Fig. 2b, c, Supplementary Fig. 4b, c). As positively charged arginine side chains often interact with the backbone of nucleic acids^14^, it is possible that increased DNA-binding affinity of the mutants helps pull the target strand toward the RuvC active site and promote DNA cleavage.

As an orthogonal approach, we sought to address the temperature dependence of target-strand cleavage. One common attribute of cold-adapted enzymes is the presence of surface-exposed glycine residues, which can increase protein flexibility and enzymatic activity by acting locally near a catalytic site^15^ or through allosteric mechanisms^16^. We generated glycine substitutions at 66 surface-exposed residues and again tested for indel activity at two target sites. Strikingly, we observed over twofold improvement relative to wild type with the E837G variant, a position that is located between the guide RNA:DNA duplex and the RuvC active site (Fig. 2d, e). Testing combinations of mutations led to progressively active variants with a final BhCas12b v4 mutant containing K846R/S893R/E837G that exhibited the highest activity across multiple targets (Fig. 2f, g, Supplementary Fig. 4d–f). In agreement with these results in human cells, purified BhCas12b v4 protein exhibited increased dsDNA cleavage activity at 37 °C and a clear reduction of nicked dsDNA (Fig. 2h, Supplementary Fig. 4g–j).

### BhCas12b v4 mediates genome editing in human cells

**Fig. 3:**
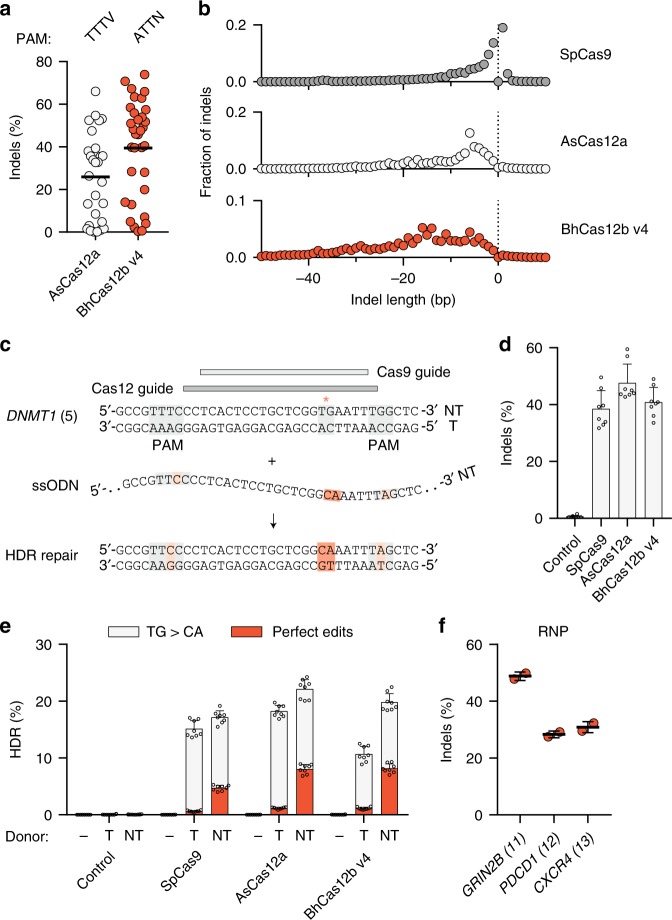
BhCas12b v4 mediates genome editing in human cells. **a** Indel activity in 293T cells of AsCas12a at 28 TTTV targets and BhCas12b v4 at 33 ATTN targets. Each dot represents a single target site, averaged from *n* = 4 replicates. **b** Average indel length during genome editing with 30 active BhCas12b guides, 45 active AsCas12a guides, and 39 active SpCas9 guides. **c** Schematic of a *DNMT1* region targetable by SpCas9 and Cas12a/b nucleases and a 120-nt ssODN donor containing a TG-to-CA mutation and PAM-disrupting mutations. **d** Indel activity of each nuclease at the *DNMT1* locus. Error bars represent s.d. from *n* = 8 replicates. **e** Frequency of homology-directed repair (HDR) using a target strand (T) or non-target strand (NT) donor. Gray bars indicate the frequency of TG-to-CA mutation, while red bars indicate perfect edits with no detectable mutations in the 36-nt sequence shown in panel **c**. Error bars represent s.d. from *n* = 6 replicates. **f** Indel activity in CD4+ human T cells following BhCas12b v4 RNP delivery. Each dot represents an individual electroporation (*n* = 2). Source data are provided as a Source Data file

Robust genome editing tools should be effective and specific across a range of targets, and therefore, we investigated Cas12b activity more thoroughly in comparison to previously studied Cas nucleases. We tested BhCas12b v4 at 56 targets across five genes in 293T cells and found robust cleavage at ATTN PAMs using AsCas12a at TTTV PAMs as a positive control (Fig. 3a). We also observed high BhCas12b v4 activity at a subset of TTTN and GTTN PAMs, although this activity was less robust than activity at ATTN PAMs (Supplementary Fig. 5a). Analysis of ATTN prevalence in the human genome revealed a similar number of targetable sites, as can be achieved with Cas12a enzymes (Supplementary Fig. 5b). In contrast to SpCas9 and AsCas12a, analysis of the indel patterns formed by BhCas12b revealed prominent larger deletions of 5–15 bp (Fig. 3b). Co-transfection of BhCas12b v4 with single-strand oligonucleotide (ssODN) donors led to comparable editing efficiency as SpCas9 and AsCas12a at a TTTC PAM target (Fig. 3c–e), and higher editing efficiency than SpCas9 at an ATTC PAM target (Supplementary Fig. 5c–e). To further evaluate the efficacy of BhCas12b v4 in human cells, we tested the ability of Cas12b ribonucleoproteins (RNPs) to edit primary human T cells. We generated BhCas12b v4-sgRNA complexes and delivered them into human CD4+ T cells by electroporation. BhCas12b v4 RNPs exhibited indel rates of 32–49% across three tested targets (Fig. 3f). Together, these data indicate that BhCas12 v4 can be harnessed as an effective programmable nuclease in a variety of genome editing contexts, including in a therapeutically relevant human cell type.

### BhCas12b v4 is a highly specific nuclease

**Fig. 4:**
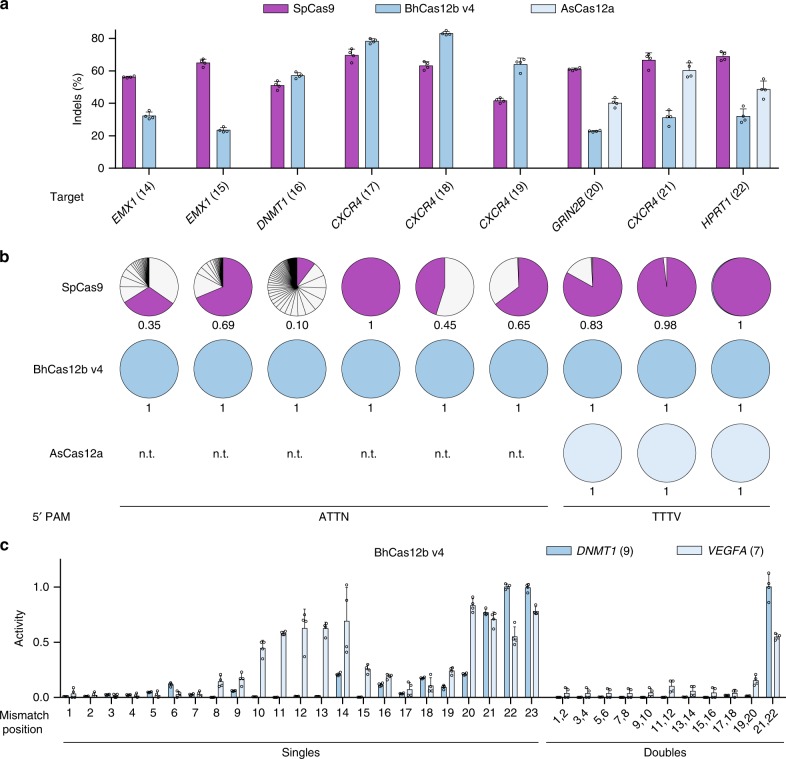
BhCas12b v4 is a highly specific nuclease. **a** Comparison of Cas9, Cas12b, and Cas12a indel activity in 293T cells at nine target sites (except for Cas12a, which was only tested at the three TTTV PAM sites) selected for Guide-Seq analysis. Error bars represent s.d. from *n* = 4 replicates. **b** Guide-Seq analysis showing the number and relative proportion of detected cleavage sites for each nuclease. Off-targets are shown as light gray wedges, while the on-target site is highlighted in purple (for SpCas9), dark blue (for BhCas12b v4), or light blue (for AsCas12a) with the fraction of on-target reads shown below. Off-targets were only detected with SpCas9. n.t., not tested. See Supplementary Fig. 6 for full analysis. **c** BhCas12b indel activity in 293T cells when mismatches are present between the guide sgRNA and target DNA. Mismatches were inserted in the sgRNA to match the target strand (i.e., C to G, A to T). Error bars represent s.d. from *n* = 4 replicates. Source data are provided as a Source Data file

We next sought to determine the genome-wide targeting specificity of BhCas12b in human cells. We chose nine target sites with comparable indel activity between different Cas nucleases (Fig. 4a) and performed Guide-Seq^17^ analysis. We did not detect any off-target sites for BhCas12b v4 or AsCas12a, whereas SpCas9 led to prominent off-target cleavage in six of the nine tested guides (Fig. 4b, Supplementary Fig. 6), consistent with other reports^18,19^. For example, for *DNMT1* (target 16), we detected 101 insertion sites with SpCas9, with only 10% of reads mapping to the target site, but no off-target sites with BhCas12b v4. Additional Guide-Seq experiments at unmatched target sites detected off-target cleavage at only 2 of 14 sites for BhCas12b v4 (Supplementary Fig. 7 and 8). Consistent with these findings, we observed limited indel activity with double mismatches between the guide RNA and target DNA in positions 1–20, and even a low tolerance for single mismatches (Fig. 4c). These results agree with the reported specificity of AacCas12b in vitro^20^ and suggest a molecular explanation for the low off-target activity observed in cells.

## Discussion

Here, we describe a member of the type-V CRISPR–Cas12b family from *Bacillus hisashii* that is suitable for genome editing in human cells. Following our work, we also identified a second ortholog from *Bacillus sp. V3–13*^21^ (41% sequence identical to BhCas12b) that also mediates genome editing in human cells (Supplementary Fig. 9a–i). We observed only a weak correlation between the activities of BhCas12b v4 and BvCas12b at matched sites (*R*^2^ = 0.48), and numerous targets were more efficiently cleaved by one of the two nucleases (Supplementary Fig. 9j). These findings emphasize the benefit of multiple orthologs and the continued need to thoroughly investigate the targeting rules of Cas nucleases. Of note, AaCas12b has also recently been shown to mediate genome editing in mammalian cells^22^, and we anticipate that continued exploration of the Cas12b family will uncover new functional members. Our engineering of BhCas12b led to a substantial increase in the efficiency of dsDNA cleavage and provides a road map for unlocking the potential of other CRISPR nucleases as genome editing tools. BhCas12b is a comparatively compact protein (at 1108 amino acids), and therefore, in contrast to SpCas9 and AsCas12a, is suitable for efficient packaging into adeno-associated virus. In combination, the small size and high-target specificity of BhCas12b v4 make it a promising tool for in vivo genome editing.

## Methods

### Generation of Cas12b expression plasmids

Cas12b loci were synthesized and cloned into pACYC184 (Genewiz) for expression in *E. coli*. The Cas12b open-reading frame (ORF) was codon optimized for human expression, while upstream and downstream sequences flanking the ORF were left unchanged. CRISPR arrays were shortened to three direct repeats and the first endogenous spacer was replaced with the FnCpf1 protospacer 1 (FnPSP1) sequence (GAGAAGTCATTTAATAAGGCCACTGTTAAAA).

### PAM discovery

*E. coli* cells expressing pACYC184-Cas12b systems were made competent with the Z-competent kit (Zymo Research). Cells expressing pACYC184-Cas12b or empty pACYC184 were transformed with a PAM library with randomized 8N sequence on the 5′ side of the FnPSP1 target site and grown overnight for 16 h. Plasmid DNA was isolated, and the library was sequenced using a 75-cycle NextSeq kit (Illumina). PAM representation in the library was determined using a custom Python script (available upon request) and compared between Cas12b and control with two independent replicates. We were unable to identify any sequence preference 7 and 8 bp upstream of the spacer, and the presented data reflect a condensed 6N library. Sequence motifs were generated using the Weblogo tool (http://weblogo.berkeley.edu). PAM wheels were generated using Krona plots (https://github.com/marbl/Krona/wiki)^23^.

### Bacterial RNA sequencing

Briefly, RNA was prepared from *E. coli* lysates using TRIzol followed by homogenization with a BeadBeater (BioSpec Products). rRNA was removed with the Ribo-Zero kit (Illumina) and libraries prepared using the NEBNext Small RNA Library Kit for Illumina (NEB). Libraries were sequenced with a 2 × 150 paired-end MiSeq run (Illumina) and the reads aligned and analyzed with Geneious R9 (Biomatters).

### Purification of Cas12b protein

Cas12b genes were cloned into bacterial expression plasmids (T7-TwinStrep-SUMO-NLS-Cas12b-NLS-3xHA) and expressed in BL21(DE3) cells (NEB #C2527H) containing a pLysS-tRNA plasmid (from Novagen #70956). Cells were grown in Terrific Broth to mid-log phase and the temperature lowered to 20 °C. Expression was induced at 0.6 OD with 0.25 mM IPTG for 16–20 h before harvesting and freezing cells at –80 °C. Cell paste was resuspended in lysis buffer (50 mM TRIS, pH 8, 500 mM NaCl, 5% glycerol, and 1 mM DTT) supplemented with EDTA-free complete protease inhibitor (Roche). Cells were lysed using a LM20 microfluidizer device (Microfluidics) and cleared lysate bound to Strep-Tactin Superflow Plus resin (Qiagen). The resin was washed using lysis buffer and Cas12b protein eluted with lysis buffer supplemented with 5 mM desthiobiotin. The TwinStrep-SUMO tag was removed by overnight digest at 4 °C with homemade SUMO protease Ulp1 at a 1:100 weight ratio of protease to Cas12b. Cleaved Cas12b protein was diluted to 200 mM NaCl and purified using a HiTrap Heparin HP column on an AKTA Pure 25L (GE Healthcare Life Sciences) with a 200 mM–1 M NaCl gradient. Fractions containing Cas12b were pooled and concentrated and loaded onto a Superdex 200 Increase column (GE Healthcare Life Sciences) with a final storage buffer of 25 mM TRIS, pH 8, 500 mM NaCl, 5% glycerol, and 1 mM DTT. Purified Cas12b protein was concentrated to 5 or 73 µM stocks and flash-frozen in liquid nitrogen before storage at –80 °C.

### In vitro RNA synthesis

All RNA was generated by annealing a DNA oligonucleotide containing the reverse complement of the desired RNA with a short T7 oligonucleotide. In vitro transcription was performed using the HiScribe T7 High Yield RNA synthesis kit (NEB) at 37 °C for 8–12 h and RNA was purified using Agencourt AMPure RNA Clean beads (Beckman Coulter).

### In vitro cleavage reactions

DNA substrates were generated by PCR amplification of pUC19 plasmids containing the FnPSP1 target site. Typical reactions contained 100 ng of DNA substrate, 250 nM of Cas12b protein, 500 nM of RNA, and a final 1× reaction buffer of 20 mM TRIS, pH 6.5, and 6 mM MgCl_2_. Reactions were quenched with 20 mM EDTA, RNA digested with 5 µg of RNAse A (Qiagen) at 37 °C for 5 min, and DNA products purified using a PCR cleanup kit (Qiagen). Reactions were run on Novex 10% TBE PAGE gels in 1× TBE buffer (Thermo Fisher Scientific) and stained with SYBR Gold (Thermo Fisher Scientific). Labeled DNA substrates were generated with IR700 and IR800-conjugated DNA oligonucleotides (IDT). For denaturing gels, DNA was mixed with an equal volume of 100% formamide followed by heat denaturation at 95 °C for 5 min. Products were separated on Novex Urea-PAGE gels (Thermo Fisher Scientific) in 1x TBE buffer preheated to 60 °C and were imaged on an Odyssey CLx device (LI-COR). Where applicable, quantitation of DNA cleavage or nicking was determined by the formula, 100 × (1 − sqrt(1 − (b + c)/(a + b + c))), where a is the integrated intensity of the undigested product and b and c are the integrated intensities of each cleavage or nicking product.

### Mammalian constructs and mutagenesis

Cas12b genes were amplified from their corresponding pACYC184 plasmids and cloned into pCDNA3.1 containing N- and C-terminal NLS tags and a C-terminal 3×HA tag. Guide expression plasmids were generated by cloning sgRNA scaffolds containing two inverted BsmBI Type IIS restriction sites behind the U6 promoter. Guides were cloned into the scaffolds by Golden Gate assembly with two annealed complementary oligonucleotides. Unless noted, all guides were 23 nt in length. See Supplementary Table 1 for guide sequences. Desired Cas12b mutations were ordered on oligonucleotides to generate two overlapping Cas12b PCR products which were assembled using Gibson Assembly Master Mix (NEB).

### Cell culture and transfections

HEK293T cells (ATCC) were cultured in Dulbecco’s modified Eagle medium with high glucose, sodium pyruvate, and GlutaMAX (Thermo Fisher Scientific), 1× penicillin–streptomycin (Thermo Fisher Scientific), and 10% fetal bovine serum (Seradigm). Cells were maintained at a confluency below 90% and tested negative for mycoplasma using the MycoAlert detection kit (Lonza). For indel analysis, 96-well plates were seeded with 17,500 cells/well approximately 16 h before transfection for a confluency of approximately 75% at the time of transfection. Each 96-well was transfected with 100 ng of nuclease-expressing plasmid and 100 ng of guide plasmid in 20 µL of Opti-MEM (Thermo Fisher Scientific) with 0.6 µL of TransIt-LT1 transfection reagent (Mirus). Cells were harvested 72 h post-transfection with QuickExtract DNA extraction solution (Lucigen).

For homology-directed repair experiments, 100 ng of nuclease, 100 ng of guide, and 100 ng of ssODNs were transfected per 96-well with 0.9 µL of TransIt-LT1 transfection reagent (Mirus). ssODNs were ordered as Ultramer DNA oligonucleotides (IDT) and contained three phosphorothioate modifications on each end.

### Deep sequencing of indel mutations

Targeted indel analysis was performed by amplifying genomic regions of interest with NEBNext High-Fidelity 2× PCR Master Mix (NEB) using a two-round PCR strategy to add Illumina P5 adaptors and unique sample-specific barcodes. Libraries were sequenced with 1 × 200-cycle MiSeq runs (Illumina). Indel rates were measured using Outknocker 2^24^.

(http://www.outknocker.org/outknocker2.htm).

### Off-target analysis

Off-target cleavage sites were identified using Guide-Seq^17^ with modified library preparation. Briefly, cells were transfected in 96-well plates with 75 ng of nuclease plasmid, 25 ng of guide plasmid, and 100 ng of annealed dsDNA oligos in 50 µL of Opti-MEM with 0.5 µL of GeneJuice Transfection Reagent (Millipore).

F: /5phos/G*T*TGTGAGCAAGGGCGAGGAGGATAACGCCTCTCTCCCAGCGACT*A*T R: /5phos/A*T*AGTCGCTGGGAGAGAGGCGTTATCCTCCTCGCCCTTGCTCACA*A*C

Cells were harvested after 72 h and 10 wells were pooled for each experiment. 1E6 cells were lysed and genomic DNA was tagmented with Tn5 followed by purification using a plasmid mini-prep column (Qiagen). Libraries were prepared using two rounds of PCR amplification with KOD Hot Start DNA Polymerase (Millipore) using a Tn5 adapter-specific primer and nested primers within the DNA donor. Libraries were sequenced with a 75-cycle NextSeq kit (Illumina). Reads were mapped to the human genome (hg38) using BrowserGenome.org^25^.

### T-cells culture

Human CD4+ T cells (STEMCELL Technologies) were cultured in RMPI 1640 (Glutamax Supplement, Gibco) supplemented with 5 mM HEPES, pH 8.0 (Gibco), 50 µg/mL penicillin/streptomycin (Gibco), 50 µM 2-mercaptoethanol (Sigma-Aldrich), 5 mM MEM nonessential amino acids (Gibco), 5 mM sodium pyruvate (Gibco), and 10% FBS (Seradigm). Cells were activated for 5–7 days post-thaw by plating every 2 days on dishes coated with 10 µg/mL of anti-CD3 (UCHT1, eBioscience, Invitrogen) and anti-CD28 (CD28.2, eBioscience, Invitrogen) monoclonal antibodies.

### RNP complexing and delivery

BhCas12b sgRNA was synthesized with three 2′O-methyl modifications at 3′ end (Integrated DNA Technologies). RNPs were formed by incubating 10 mg/mL protein with 50 µM annealed RNA at a 1:1 molar ratio at 37 °C for 15 min. RNPs were stored on ice until electroporation.

Cells were electroporated using the Amaxa P3 Primary Cell 4D-Nucleofector X Kit (Lonza). Per reaction, 3 × 10^5^ stimulated CD4+ T cells were pelleted and resuspended in 20 µL of P3 buffer. In total, 4.5 µM Cas9 or Cas12b protein, precomplexed with crRNA and tracrRNA, was added and the mixture transferred to the electroporation cuvette. Cells were electroporated using program EH-115 on the Amaxa 4D-Nucleofector (Lonza). Eighty microliters of prewarmed complete media was immediately added to the cells post pulse and the cells were incubated at 37 °C for 30 min to recover in the cuvette. After recovery, 50 µL of the cell suspension was added to 50 µL of complete medium plus 80 IU/mL IL-2 (STEMCELL Technologies) for a final concentration of 40 IU/mL IL-2. The cells were plated on CD3/CD28 precoated 96-well plates. Cells were harvested after 48 h for indel analysis.

### Reporting summary

Further information on experimental design is available in the Nature Research Reporting Summary linked to this article.

### Code availabilty

Custom scripts are available upon request.

## Supplementary information

Supplementary Information

Peer Review File

Reporting Summary

Source Data

## Data Availability

Sequencing data has been uploaded to the Sequence Read Archive under Bioproject accession code PRJNA510943. All other data are available from the authors upon reasonable request.
