# Supplementary material for: Engineering of CRISPR-Cas12b for human genome editing
Source: Nat Commun. 2019 Jan 22;10:212. doi: 10.1038/s41467-018-08224-4 (PMC6342934; doi:10.1038/s41467-018-08224-4)
Supplement: Supplementary file 1 — Supplementary Information [file 41467_2018_8224_MOESM1_ESM.pdf]

Engineering of CRISPR-Cas12b for human genome editing

Strecker et al.

Supplementary Figure 1. PAM discovery of Cas12b orthologs

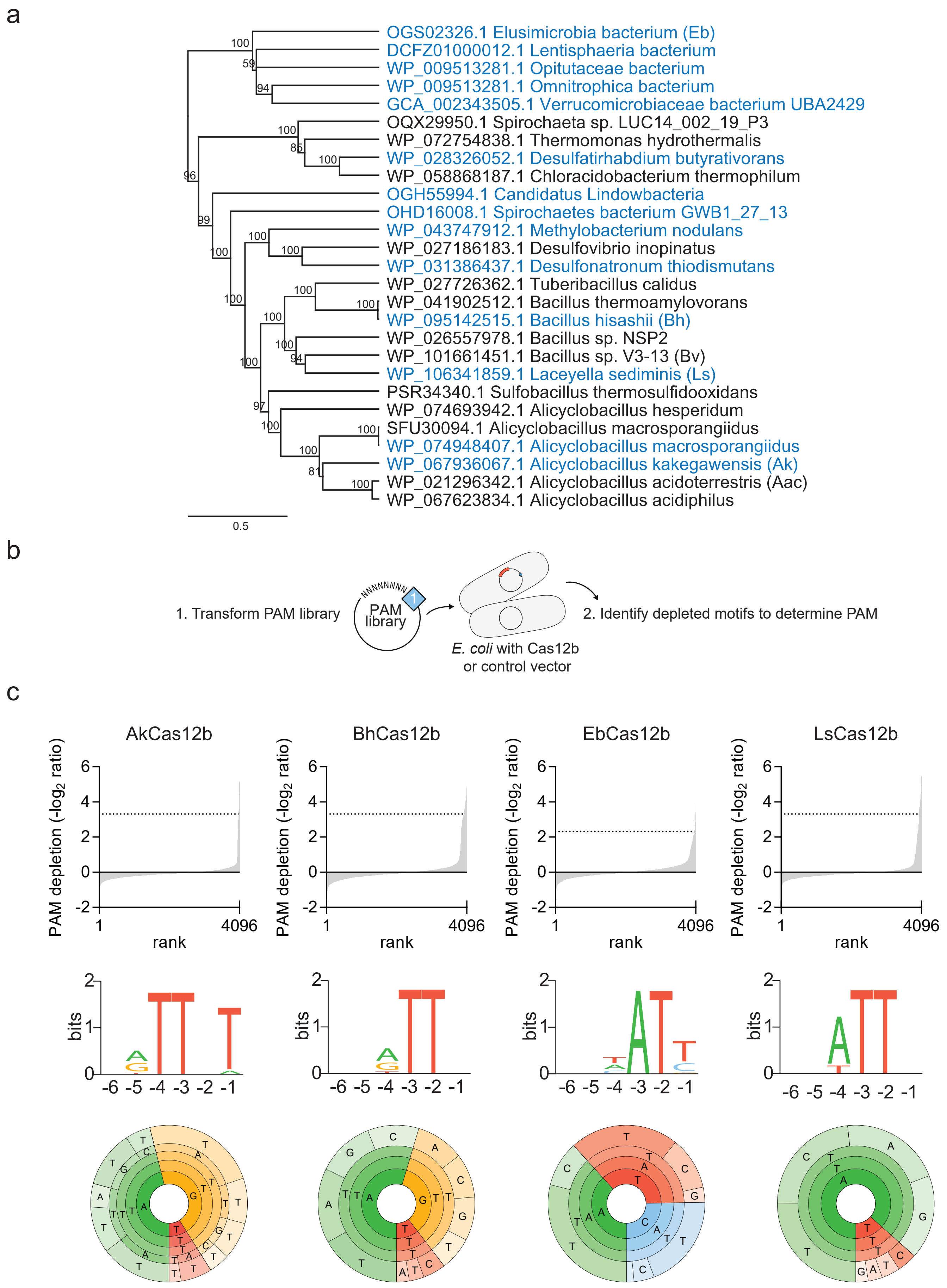

Supplementary Figure 1. PAM discovery of Cas12b orthologs

a) Phylogenetic tree of the subtype V-B effector Cas12b proteins. Sequences are denoted by Genbank protein accession number and species name. The proteins that were experimentally studied in this work are highlighted in blue. **b**) Schematic of the PAM discovery assay in *E. coli*[1,9]. **c**) Depleted PAMs were detected in only 4 out of 14 Cas12b systems in *E. coli.* A depletion threshold was set at a $-\log_2$ ratio of 3.32 (dotted line) except for EbCas12b, which had a threshold set at 2.32. Depleted PAMs are shown as sequence motifs as well as PAM wheels[23] starting in the middle of the wheel for the first 5′ base exhibiting sequence information.

Supplementary Figure 2. Cas12b RNA-Seq and in vitro reconstitution

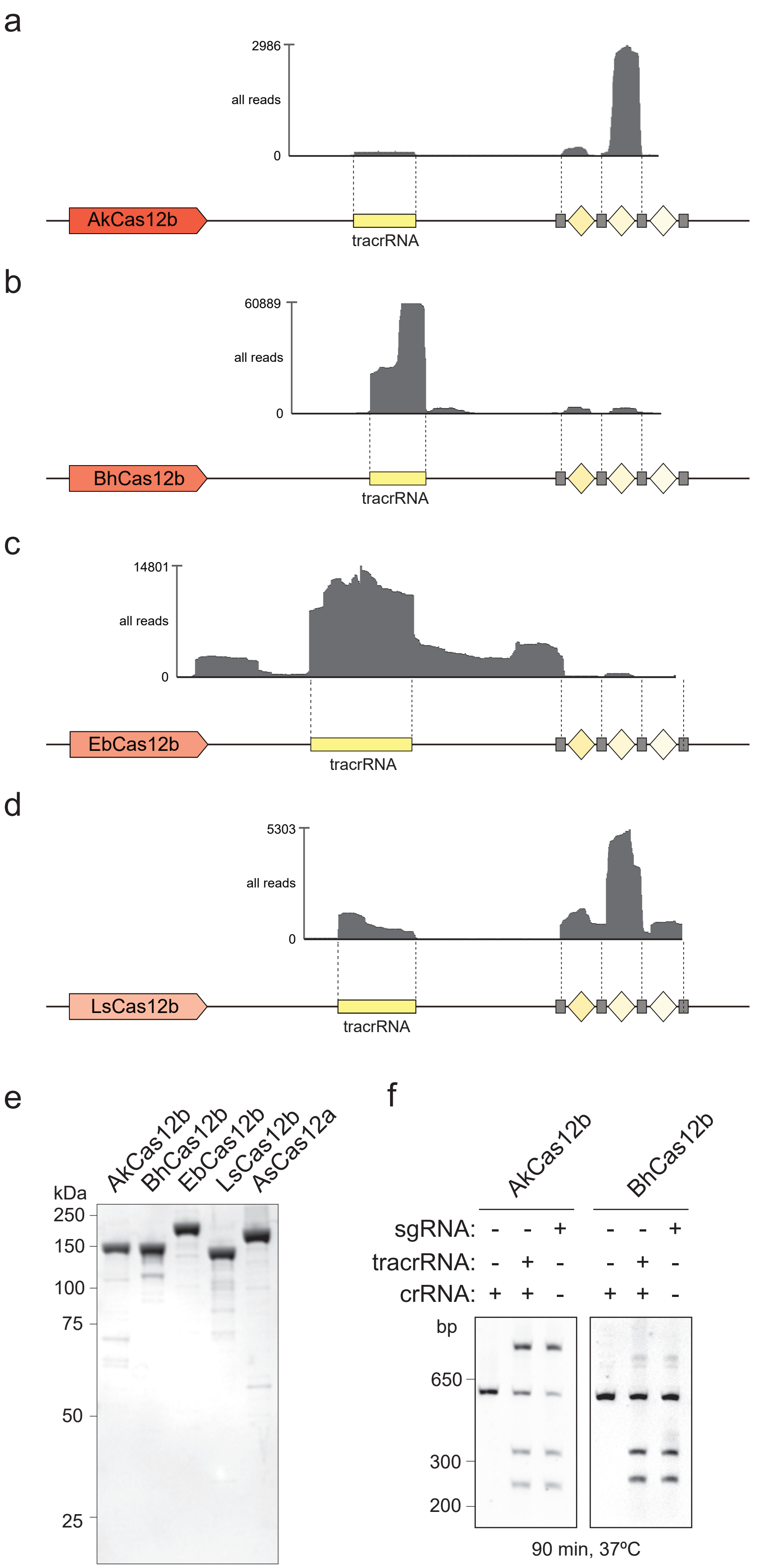

Supplementary Figure 2. Cas12b RNA-Seq and in vitro reconstitution

a-d) Alignment of small RNA-Seq reads for AkCas12b, BhCas12b, EbCas12b, and LsCas12b. The location of the tracrRNA used in cleavage reactions is highlighted in yellow. **e**) Coomassie stained SDS-PAGE gel of purified Cas12b proteins used in this study and commercially produced AsCas12a (IDT). **f**) In vitro cleavage reactions with AkCas12b and BhCas12b comparing tracrRNA and crRNA to v1 sgRNA scaffolds.

Supplementary Figure 3. Cas12b sgRNA optimization in mammalian cells

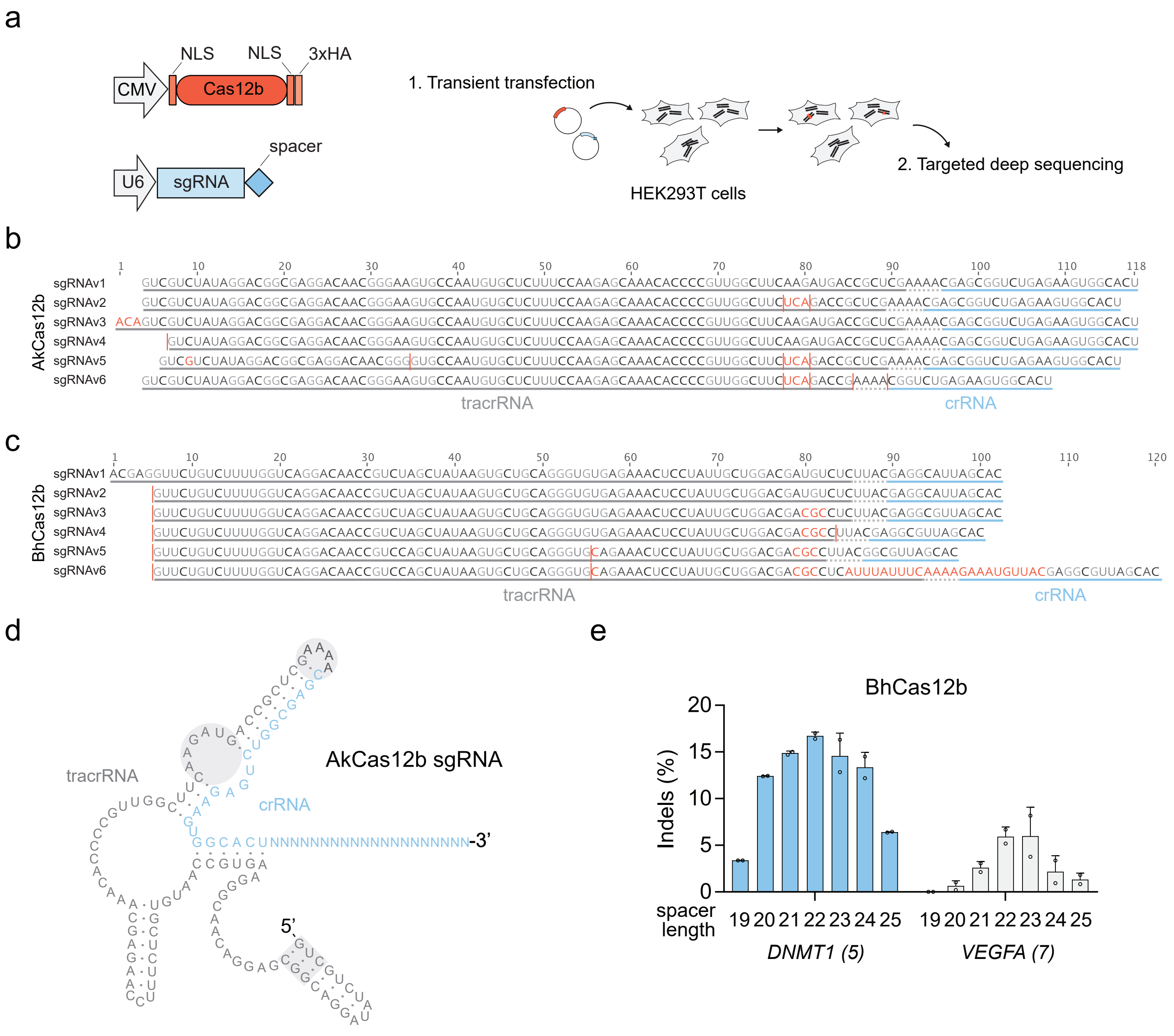

Supplementary Figure 3. Cas12b sgRNA optimization in mammalian cells

a) Schematic of expression constructs and assay for indel activity in mammalian cells. **b,c**) Sequence of AkCas12b and BhCas12b sgRNA variants. The location of deletions is denoted with a red line. **d**) Schematic of AkCas12b sgRNA design 1 with grey shading highlighting the location of changes. **e**) Indel activity in 293T cells with BhCas12b and varying spacer lengths. Error bars represent s.d. from n=2 replicates. Source data are provided as a Source Data file.

Supplementary Figure 4. Rational engineering of BhCas12b

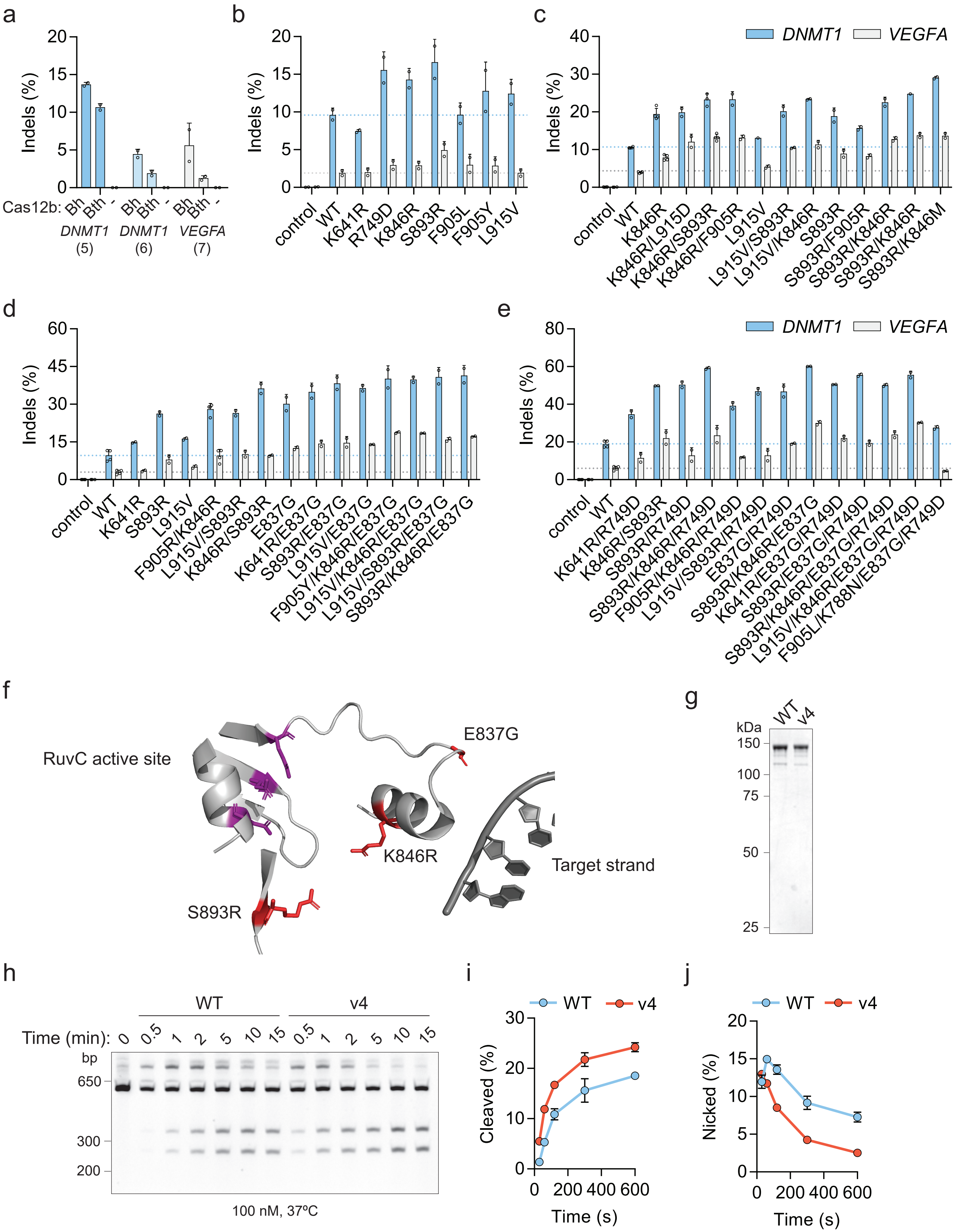

Supplementary Figure 4. Rational engineering of BhCas12b

a) Comparison of indel activity between BhCas12b and the highly similar BthCas12b in 293T cells. Error bars represent s.d. from n=2 replicates. **b-e**) Indel activity of BhCas12b mutant combinations at *DNMT1* (target 5) and *VEGFA* (target 7). Error bars represent s.d. from a minimum of n=2 replicates. **f**) BhCas12b v4 mutations modeled into the structure of BthCas12b (PDB:5wti [10.2210/pdb5WTI/pdb]) using Pymol (Schrodinger). **g**) Coomassie stained SDS-PAGE gel of purified BhCas12b WT and v4 protein. **h**) In vitro cleavage time-course with BhCas12b WT and v4 variant. Gel is representative image from n=3 experiments. **i,j**) Quantitation of dsDNA cleavage products (**i**) and upper nicked product (**j**) from the reactions shown in panel **h**. Error bars represent s.d. from n=3 experiments. Source data are provided as a Source Data file.

Supplementary Figure 5. BhCas12b v4 mediates genome editing in human cell lines

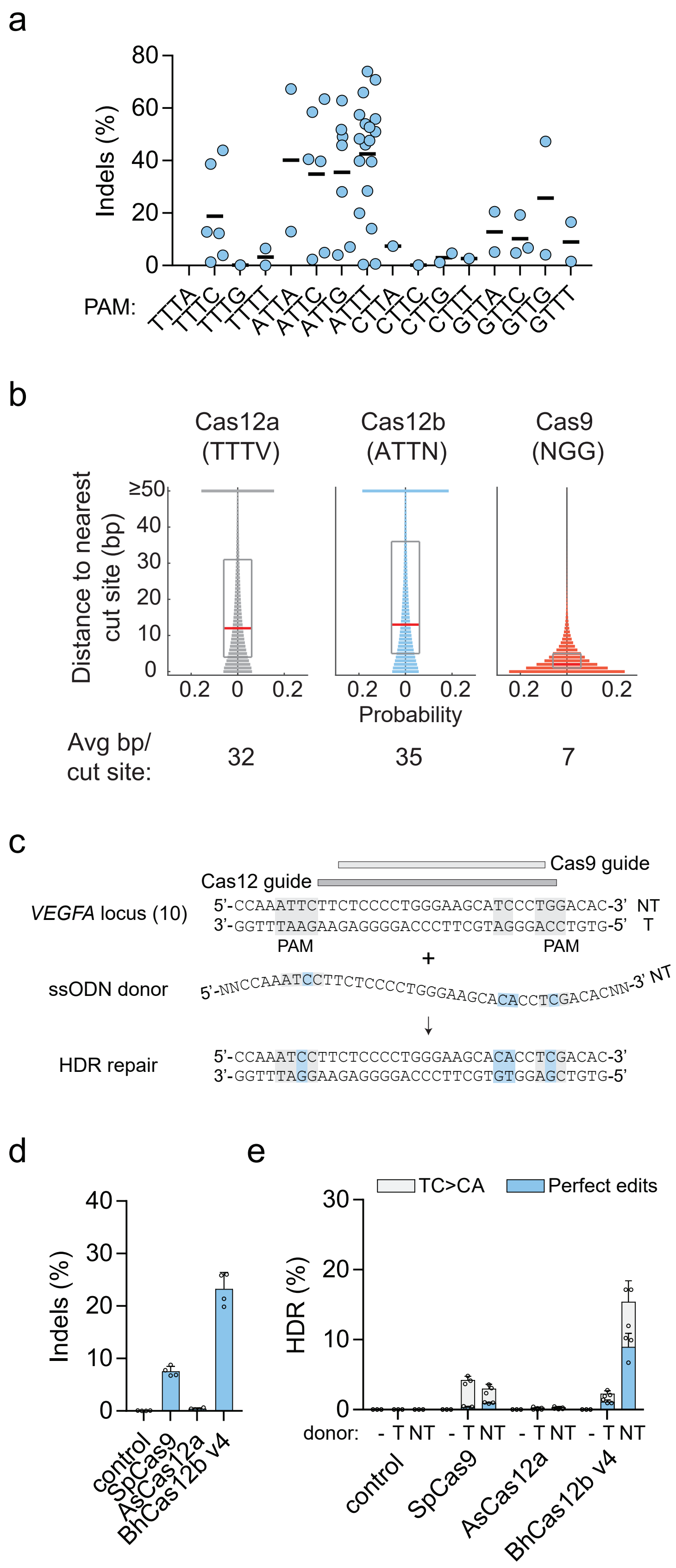

Supplementary Figure 5. BhCas12b v4 mediates genome editing in human cells lines

a) BhCas12b v4 indel activity in 293T cells at 56 targets. Each dot represents a single target site, averaged from n=4 replicates. **b**) Analysis of PAM prevalence for Class 2 CRISPR-Cas nucleases. Probability mass function for the distance from each base within non-masked human coding sequences to the nearest Cas9 or Cas12 cleavage site. **c**) Schematic of a *VEGFA* (target 10) site targetable by SpCas9 and Cas12b nucleases and a 120-nt ssODN donor containing a TC to CA mutation and PAM disrupting mutations **d**) Indel activity of each nuclease at the locus. Error bars represent s.d. from n=3 replicates. **e**) Frequency of homology-directed repair (HDR) using a target strand (T) or non-target strand (NT) donor. Grey bars indicate the frequency of TC to CA mutation, while blue bars indicate perfect edits with no detectable mutations in the 36-nt sequence shown in panel **c**. Error bars represent s.d. from n=3 replicates. Source data are provided as a Source Data file.

Supplementary Figure 6. Specificity analysis of matched CRISPR-Cas nuclease targets

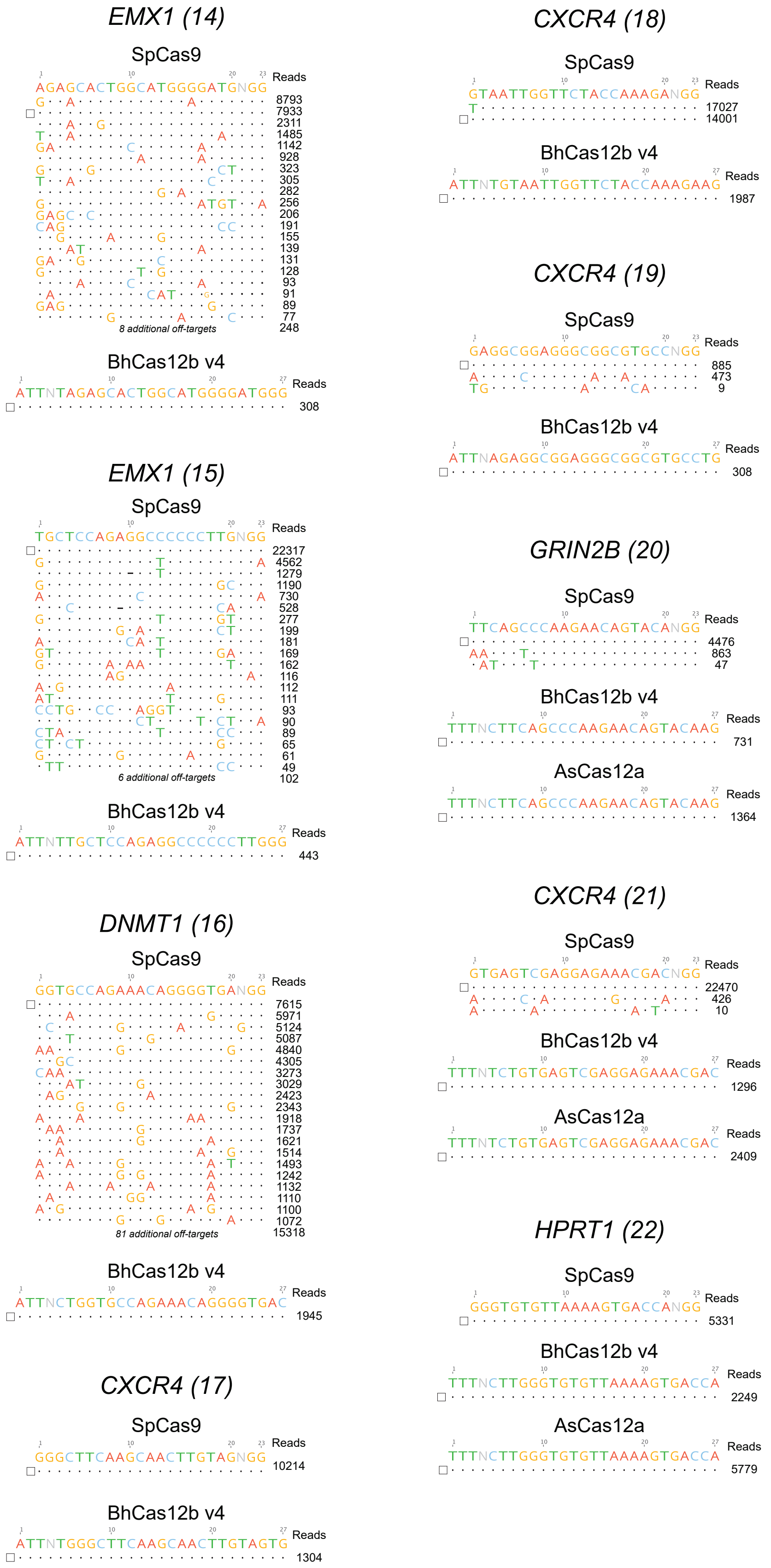

Supplementary Figure 6. Specificity analysis of matched CRISPR-Cas nuclease targets

Full Guide-Seq analysis of detected off-targets in Fig. 4b. A list of detected cleavage sites (up to 20 per target) is presented for each nuclease with the on-target site denoted with a small box. Mismatches to the guide sequence are highlighted.

Supplementary Figure 7. Specificity analysis of unmatched CRISPR-Cas nuclease targets

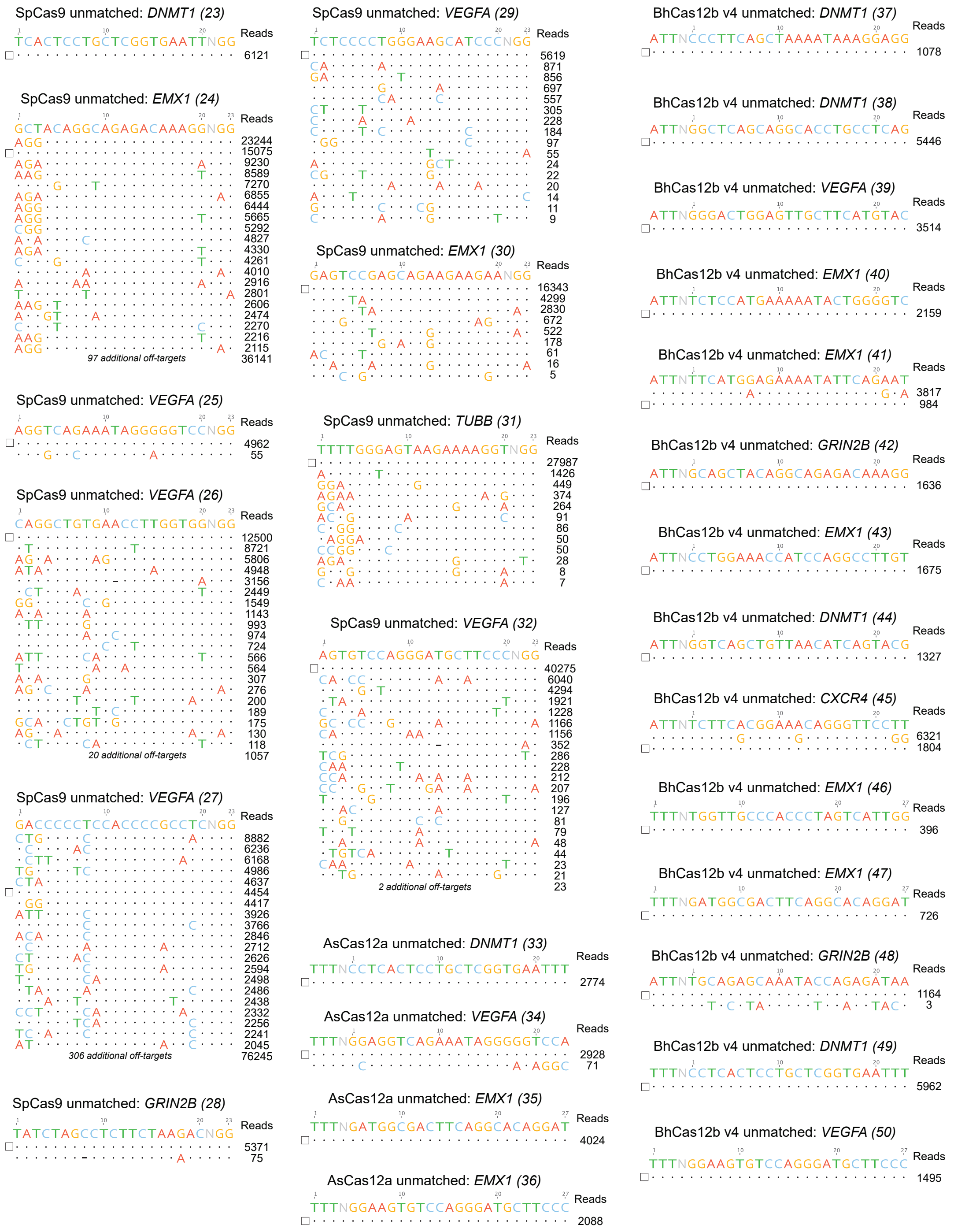

Supplementary Figure 7. Specificity analysis of unmatched CRISPR-Cas nuclease targets

Full Guide-Seq analysis of detected off-targets for unmatched targets. A list of detected cleavage sites (up to 20 per target) is presented for each nuclease with the on-target site denoted with a small box. Mismatches to the guide sequence are highlighted.

Supplementary Figure 8. Characterization of BvCas12b

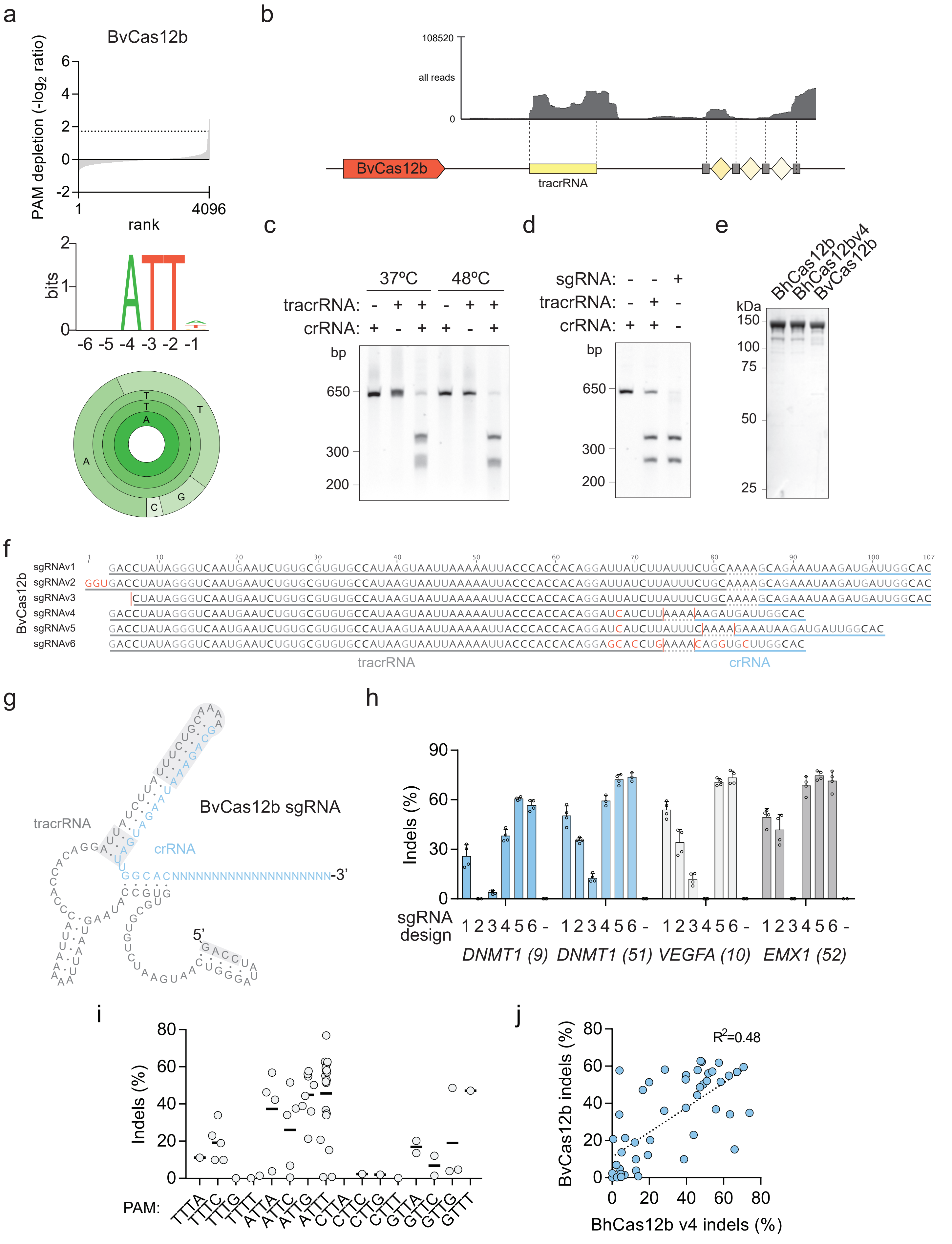

Supplementary Figure 8. Characterization of BvCas12b

a) PAM discovery as described in Supplementary Fig. 1b,c. **b**) Alignment of small RNA-Seq reads for BvCas12b. The location of the tracrRNA used in cleavage reactions is highlighted in yellow. **c,d**) In vitro reactions with 250 nM purified BvCas12 protein and synthesized RNA were carried out at the indicated temperatures for 90 min. **e**) Coomassie stained SDS-PAGE gel of purified BvCas12b. **f**) BvCas12b sgRNA variants. The location of deletions is denoted with a red line. **g**) Schematic of BvCas12b sgRNA design 1 with grey shading highlighting the location of changes to the guide design in variants 2 - 6. **h**) BvCas12b indel activity in 293T cells with sgRNA variants. Error bars represent s.d. from n=4 replicates. **i**) BvCas12b indel activity in 293T cells at 57 targets. Each dot represents a single target site, averaged from n=4 replicates. **j**) Correlation of BhCas12b v4 and BvCas12b activity at matched target sites. Source data are provided as a Source Data file.

Supplementary Figure 9. BvCas12b mismatch tolerance and specificity

a

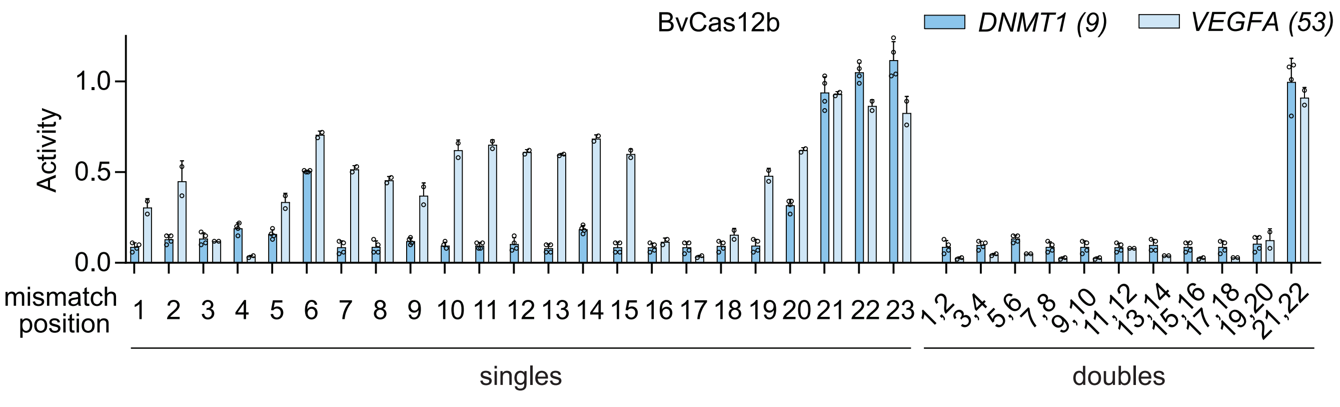

b

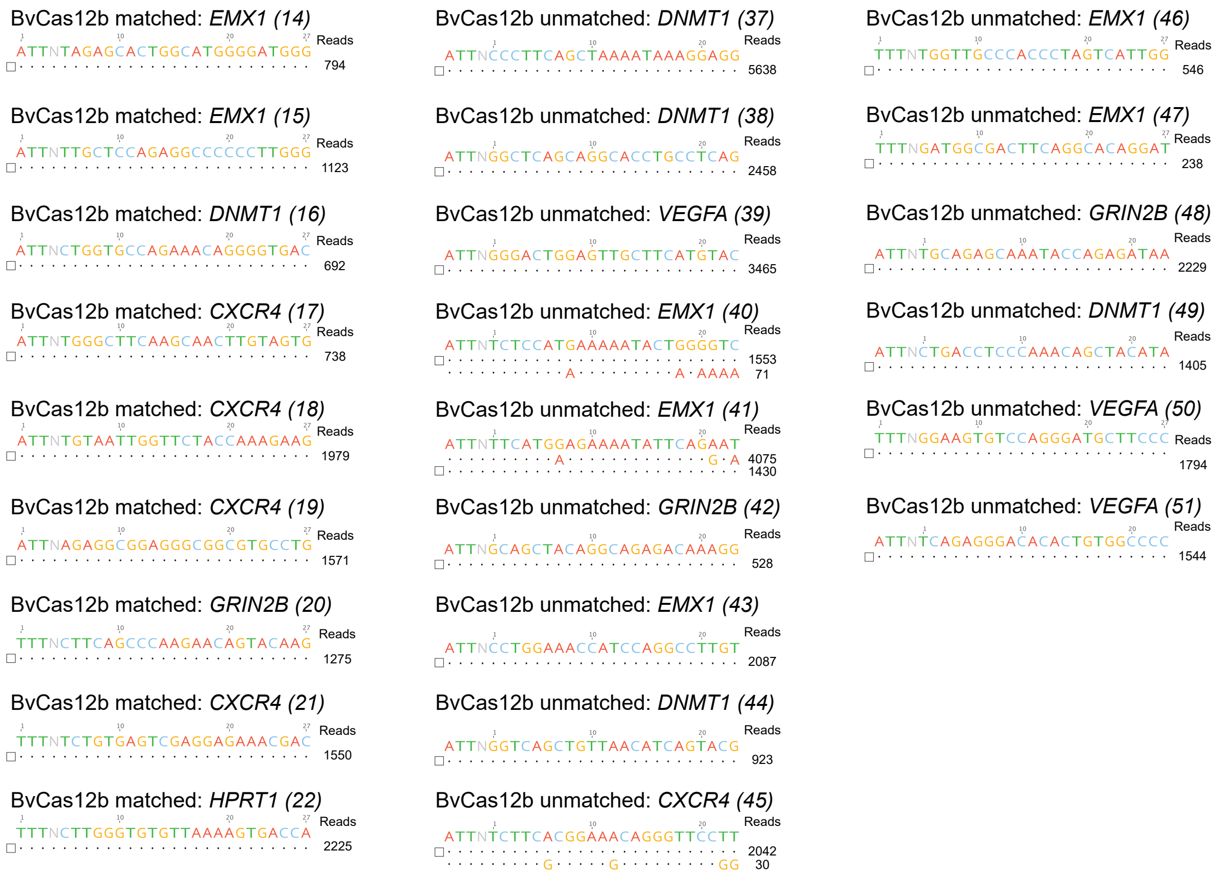

Supplementary Figure 9. BvCas12b mismatch tolerance and specificity

a) BvCas12b indel activity in 293T cells when mismatches are present between the guide sgRNA and target DNA. Mismatches were inserted in the sgRNA to match the target strand (i.e., C to G, A to T). Error bars represent s.d. from n=4 replicates. **b**) Guide-Seq analysis of BvCas12b at 24 targets. A list of detected cleavage sites is presented with the on-target site denoted with a small box. Mismatches to the guide sequence are highlighted. Source data are provided as a Source Data file.

Supplementary Table 1: Guide sequences used in this study

Gene	Target	5' PAM	Sequence
DNMT1	1	GTTCT	AGACCCAGAGGCTCAAGTGAGCA
DNMT1	2	ATTTT	AGCTGAAGGGAAATAAAAGGAAA
VEGFA	3	ATTCT	TCTCCCCTGGGAAGCATCCCTGG
EMX1	4	ATTTT	TCATGGAGAAAATATTCAGAATC
DNMT1	5	TTTC	CCTCACTCCTGCTCGGTGAATTT
DNMT1	6	TTTG	AGGAGTGTTCAGTCTCCGTGAAC
VEGFA	7	TTTG	GGAGGTCAGAAATAGGGGGTCCA
VEGFA	8	TTTC	CAAAGCCCATTCCCTCTTTAGCC
DNMT1	9	ATTT	CCCTTCAGCTAAAATAAAGGAGG
VEGFA	10	ATTC	TTCTCCCCTGGGAAGCATCCCTG
GRIN2B	11	ATTC	TGCAGAGCAAATACCAGAGATAA
PDCD1	12	ATTG	CGCCGGGCCCTGACCACGCTCAT
CXCR4	13	ATTC	CCGACTTCATCTTTGCCAACGTC
EMX1	14	ATTT	TAGAGCACTGGCATGGGGATGGG
EMX1	15	ATTC	TTGCTCCAGAGGCCCCCCTTGGG
DNMT1	16	ATTC	CTGGTGCCAGAAACAGGGGTGAC
CXCR4	17	ATTC	TGGGCTTCAAGCAACTTGTAGTG
CXCR4	18	ATTT	TGTAATTGGTTCTACCAAAGAAG
CXCR4	19	ATTT	AGAGGCGGAGGGCGGCGTGCCTG
GRIN2B	20	TTTC	CTTCAGCCCAAGAACAGTACAAG
CXCR4	21	TTTC	TCTGTGAGTCGAGGAGAAACGAC
HPRT1	22	TTTC	CTTGGGTGTGTTAAAAGTGACCA
DNMT1	23	-	TCACTCCTGCTCGGTGAATT
EMX1	24	-	GCTACAGGCAGAGACAAAGG
VEGFA	25	-	AGGTCAGAAATAGGGGGTCC
VEGFA	26	-	CAGGCTGTGAACCTTGGTGG
VEGFA	27	-	GACCCCCTCCACCCCGCCTC
GRIN2B	28	-	GTATCTAGCCTCTTCTAAGAC
VEGFA	29	-	TCTCCCCTGGGAAGCATCCC
EMX1	30	-	GAGTCCGAGCAGAAGAAGAA
TUBB	31	-	TTTTGGGAGTAAGAAAAGGT
VEGFA	32	-	AGTGTCCAGGGATGCTTCCC
DNMT1	33	TTTC	CCTCACTCCTGCTCGGTGAATTT
VEGFA	34	TTTG	GGAGGTCAGAAATAGGGGGTCCA
EMX1	35	TTTG	GATGGCGACTTCAGGCACAGGAT
EMX1	36	TTTG	GGAAGTGTCCAGGGATGCTTCCC
DNMT1	37	ATTT	CCCTTCAGCTAAAATAAAGGAGG

DNMT1	38	ATTT	GGCTCAGCAGGCACCTGCCTCAG
VEGFA	39	ATTT	GGGACTGGAGTTGCTTCATGTAC
EMX1	40	ATTT	TCTCCATGAAAAATACTGGGGTC
EMX1	41	ATTT	TTCATGGAGAAAATATTCAGAAT
GRIN2B	42	ATTG	GCAGCTACAGGCAGAGACAAAGG
EMX1	43	ATTT	CCTGGAAACCATCCAGGCCTTGT
DNMT1	44	ATTG	GGTCAGCTGTTAACATCAGTACG
CXCR4	45	ATTT	TCTTCACGGAAACAGGGTTCCTT
EMX1	46	TTTG	TGGTTGCCCACCCTAGTCATTGG
EMX1	47	TTTG	GATGGCGACTTCAGGCACAGGAT
DNMT1	49	TTTC	CCTCACTCCTGCTCGGTGAATTT
VEGFA	50	TTTG	GGAAGTGTCCAGGGATGCTTCCC
DNMT1	51	ATTT	GGCTCAGCAGGCACCTGCCTCAG
EMX1	52	ATTT	TTCATGGAGAAAATATTCAGAAT
VEGFA	53	ATTT	CTGACCTCCCAAACAGCTACATA
