# Supplementary material for: Engineering of CRISPR-Cas12b for human genome editing
Source: Nat Commun. 2019 Jan 22;10:212. doi: 10.1038/s41467-018-08224-4 (PMC6342934; doi:10.1038/s41467-018-08224-4)
Supplement: Supplementary file 2 — Peer Review File [file 41467_2018_8224_MOESM2_ESM.pdf]

This manuscript has been previously reviewed at another journal that is not operating a transparent peer review scheme. This document only contains reviewer comments and rebuttal letters for versions considered at Nature Communications. Mentions of the other journal have been redacted.

Reviewers' Comments:

Reviewer #1:

Remarks to the Author:

The authors have addressed my concerns. The manuscript should be published timely.

Reviewer #2:

Remarks to the Author:

Thank you for answering my questions.

Reviewer #3:

Remarks to the Author:

In this revised manuscript by Strecker and coworkers, the authors have made a variety of modifications to the manuscript that significantly improve its clarity and flow. The authors have addressed all of my concerns and I believe the manuscript in its current form is suitable for publication in nature communications.
